# The Genetic Diversity and the Divergence Time in Extant Primitive Mayfly, *Siphluriscus chinensis* Ulmer, 1920 Using the Mitochondrial Genome

**DOI:** 10.3390/genes13101780

**Published:** 2022-10-02

**Authors:** Yao Tong, Chen-Yang Shen, Yu-Yang Zhao, Yi-Jie Lin, Lian Wu, Kenneth B. Storey, Dan-Na Yu, Jia-Yong Zhang

**Affiliations:** 1 College of Chemistry and Life Science, Zhejiang Normal University, Jinhua 321004, China; 2 Department of Biology, Carleton University, Ottawa, ON K1S 5B6, Canada; 3 Key Lab of Wildlife Biotechnology, Conservation and Utilization of Zhejiang Province, Zhejiang Normal University, Jinhua 321004, China

**Keywords:** mitochondrial genome, phylogenetic relationship, divergence time, cryptic species

## Abstract

In this study, the mitochondrial (mt) genomes of *Siphluriscus chinensis* (Ephemeroptera: Siphluriscidae) were evaluated in specimens collected from two sites in China: Niutou Mountain, Zhejiang Province (*S. chinensis* NTS) and Leigong Mountain, Guizhou Province (*S. chinensis* LGS) and were successfully sequenced. The lengths of the mt genomes of *S. chinensis* NTS and *S. chinensis* LGS were 15,904 bp (ON729390) and 15,212 bp (ON729391), respectively. However, an in-depth comparison of the two mt genomes showed significant differences between the specimens collected from the two sites. A detailed analysis of the genetic distance between *S. chinensis* NTS and *S. chinensis* LGS was undertaken to further achieve an accurate delimitation of *S. chinensis*. The genetic distance between *S. chinensis* NTS and the other three species within Siphluriscidae was a high value, above 12.2%. The two mt genomes were used to reconstruct phylogenetic relationships and estimate divergence time. The results demonstrated robust differences between *S. chinensis* NTS and *S. chinensis* LGS, which revealed that a kind of cryptic species existed. Maximum likelihood (ML) and Bayesian inference (BI) analyses produced well-supported phylogenetic trees that showed evolutionary relationships between Siphluriscidae (((*S. chinensis* HQ875717 + *S. chinensis* MF352165) + *S. chinensis* LGS) + *S. chinensis* NTS). The most recent common ancestor (MRCA) of four species within Siphluriscidae began to diversify during the Neogene [11.80 million years ago (Mya); 95% highest posterior densities (HPD) = 6.17–19.28 Mya], and *S. chinensis* NTS was first to diverge from the branches of *S. chinensis* LGS. In short, based on mitochondrial genomes, our results showed that the specimens collected from Leigong Mountain, Guizhou Province (*S. chinensis* LGS) belonged to *S. chinensis*, and the specimens collected from Niutou Mountain, Zhejiang Province (*S. chinensis* NTS) were a cryptic species of *S. chinensis*.

## 1. Introduction

As a primitive group of extant pterygote insects, Ephemeroptera (commonly known as mayflies) can be dated back to the early Permian periods or late Carboniferous [1,2]. According to recent studies, 42 families, 400 genera and more than 3500 species have been recorded within Ephemeroptera [2,3,4]. Siphluriscidae is considered to be the most primitive family within Ephemeroptera because of its plesiomorphy, but the phylogenetic position of Siphluriscidae has been a hot topic of debate [5,6,7,8,9,10]. In 1920, Ulmer first described the male imago and subimago of *Siphluriscus chinensis* collected from Guangdong Province in China and established the genus *Siphluriscus*, which was classified into Siphlonuridae [11]. In 1955, Demoulin supplemented the description of the subimago and male imago of *S. chinensis* from Ulmer [12]. Edmunds and Koss assigned *Siphluriscus*, *Acanthametropus*, *Analetris* and *Stackelbergisca* to Acanthametropodinae in 1972 [13]. In 1974, Demoulin grouped *Siphluriscus* and *Stackelbergisca* as a subfamily of Siphlonuridae [14]. In 1994, McCafferty and Wang redescribed the specimens from Ulmer, reviewed the higher classification of the genera *Siphluriscus*, *Acanthametropus* and *Analetris* and concluded that *Siphluriscus* still belonged to the family Siphlonuridae [15]. In 2003, Zhou and Peters described associated larvae and imago of *S. chinensis* collected from Zhejiang Province and established a new family, Siphluriscidae, which included an extant genus, *Siphluriscus,* and an extinct genus, *Stackelbergisca* [16]. According to Van et al., *S. chinensis* was first discovered in Vietnam [17]. As Ephemeroptera have many primitive and unique features, they are significant when discussing their relationship within Insecta [6,7]. Nevertheless, the phylogenetic relationship within Ephemeroptera has been controversial. Based on different morphological characteristics, morphological classification basis and molecular marker data analysis, the results of the phylogenetic relationship within Ephemeroptera were inconsistent [1,18,19,20,21,22,23,24].

The mitochondrion is a fundamental eukaryotic organelle that plays a significant role in the energy metabolism of eukaryotic cells [25]. The mitochondrial (mt) genome is one of the most widely used in molecular phylogenetic studies [26]. Insect mt genomes usually contain thirteen protein-coding genes (PCGs), twenty-two transfer RNAs (tRNAs), two ribosomal RNAs (rRNAs) and a single central non-coding region [25]. Due to its rapid evolution rate, maternal inheritance and small genome size, the mt genome has been extensively used as a molecular marker for reconstruction of phylogenetic relationships and identification of cryptic species [7,25,27,28,29,30,31,32]. Currently, most researchers define cryptic species as being two or more distinct species that have been misclassified (or hidden) under the same species name [33]. The mt genome is one of the best options for using individual sequence data to identify the presence of cryptic species and has been instrumental in distinguishing among closely related species [31,34,35,36,37]. Combining morphological features and molecular data can effectively distinguish differences between species and provide a reference for species delimitation [38].

At present, some studies have speculated that cryptic species may exist in Ephemeroptera due to differences in flying ability and isolation of water systems [39,40]. The currently recorded distribution of *S. chinensis* is in Vietnam and China [17], and there has been little research on species delimitation of *S. chinensis* in recent years. Therefore, it is interesting to explore the presence of cryptic species in *S. chinensis*. The present study not only successfully obtained mt genomes from two populations of *S. chinensis* from Niutou Mountain, Zhejiang Province and Leigong Mountain, Guizhou Province, China, but also reconstructed phylogenetic relationships within Siphluriscidae to explore the species delimitation of *S. chinensis*.

## 2. Materials and Methods

### 2.1. Sample Collection and Morphological Identification

Based on previous references [5,16], two locations of Niutou Mountain (28°64′ N, 119°46′ E), Zhejiang Province and Leigong Mountain (26°15′ N, 108°05′ E), Guizhou Province, China were selected as the sampling sites. The kicking net method was mainly used to capture *S. chinensis* larvae. Female larvae of *S. chinensis* from two locations were observed and photographed under an optical SMZ-1500 stereomicroscope (Nikon, Tokyo, Japan) with a TSView7 digital camera attached (Tucsen, Fujian, China) in order to focus on the mandibulate mouthparts, legs, claw, gills and caudal filaments. Using Adobe Illustrator CS4 software [41], photographs of the morphological structure from optical were further measured and analyzed. After morphological identification, the samples were deposited in the Animal Herbarium, College of Life Science and Chemistry, Zhejiang Normal University, China. This experimental design was approved by the Animal Research Ethics Committee of Zhejiang Normal University.

### 2.2. DNA Extraction, PCR Amplification and Sequencing

Total genomic DNA was isolated from muscle tissue of whole individuals using an Ezup Column Animal Genomic DNA Purification Kit (Sangon Biotech Company, Shanghai, China). Several fragments were amplified using the 13 pairs of universal primers, as described in Zhang et al. [42]. After that, we designed specific primers based on the sequenced fragments using Primer Premier 5.0 [43]. After electrophoresis and gel purification, all PCR products were sequenced bidirectionally using the primer-walking method and AB13730XL by Sangon Biotech Company (Shanghai, China). Two mt genomes of *S. chinensis* NTS and *S. chinensis* LGS were annotated and deposited in the GenBank database, with accession numbers ON729390 and ON729391, respectively.

### 2.3. Gene Annotation and Sequence Analyses

The contiguous and overlapping nucleotide fragments were manually proofread, assembled and analyzed using DNASTAR Package v.7.1 (Burland T.G., Totowa, NJ, USA) [44]. We identified the tRNA genes through the online MITOS service (http://mimitos.bioinf.uni-leipzig.de/index.py) (accessed on 17 April 2022) [45]. Two mt genomes of *S. chinensis* (HQ875717, MF352165) were downloaded from the National Center for Biotechnology Information (NCBI) (https://www.ncbi.nlm.nih.gov/) (accessed on 2 May 2022) as a reference. The amino acid sequences of the thirteen PCGs and two rRNA genes (12S and 16S rRNA) were identified and aligned using the Clustal W program of Mega v.7.0 (Sudhir K., Philadelphia, PA, USA) [46]. The Kimura-2 parameter (K2P) [47] distances program of Mega v.7.0 was implemented to calculate the pairwise genetic distances. Circular mt maps of the newly determined sequences were drawn using CG View v.1.0 (Grant J.R., Alberta, Canda) online Server (http://cgview.ca/) (accessed on 18 May 2022) [48]. Nucleotide composition, composition skewness, codon usage and relative synonymous codon usage (RSCU) of the two mt genomes were calculated by PhyloSuite v.1.2.2 (Zhang D., Wuhan, China) [49]. The AT and GC skews were calculated according to the following formulae: AT skew = (A − T)/(A + T) and GC skew = (G − C)/(G + C) [50]. The Ka/Ks ratio of the 13 PCGs was calculated by KaKs Calculator v.2 (Zhang Z., Beijing, China) [51]. 

### 2.4. Phylogenetic Analyses

Phylogenetic analyses within Ephemeroptera were performed by combining the two newly sequenced mt genomes and forty-one previously published Ephemeroptera mt genomes (Table S1), including sequences from Siphluriscidae (2), Caenidae (5), Ephemerellidae (13), Ephemeridae (5), Leptophelebiinae (4), Neoephemeridae (2), Polymitarcyidae (1), Potamanthidae (3) and Vietnamellidae (6) [5,7,8,23,52,53,54,55,56,57,58,59,60,61]. Regarding the selection of outgroups, two Odonata species (KC878732, KU958378) were downloaded from the NCBI for phylogenetic analyses [62,63]. The datasets were divided into two types, the PCG123 dataset (the first, the second and the third codons positions of the 13 PCGs) and the PCG12 dataset (the first and the second codons positions of the 13 PCGs). Based on the nucleotide sequences dataset of the 13 PCGs, the substitution saturation was tested by DAMBE v.4.2 (Xia X., Hong Kong, China) [64]. Due to the third codon positions having saturated, the first and the second codon of the 13 PCGs (the PCG12 dataset) were used for phylogenetic analyses. We used the programs MAFFT v.7 (Katoh K., Osaka, Japan) and Gblock 0.91b with default settings to align the PCG12 dataset and screen for conserved regions, respectively [65,66]. After that, the resulting alignments were concatenated in PhyloSuite v.1.2.2 (Zhang D., Wuhan, China) and format conversion used Geneious v.8.1.6 (Kearse M., Auckland, New Zealand) [49,67]. AliGROOVE at default settings was used to analyze the heterogeneity between the nucleotide sequences [68]. PartitionFinder v.2.2.1 (Lanfear R., Canberra, Australian) was employed to select the optimal partitioning schemes and the best substitution model of the first and second codon of the 13 PCGs dataset for Bayesian inference (BI) and maximum likelihood (ML) analyses [69]. A total of seven partitions were found in the PCG12 dataset, and the results are displayed in Table S2. The GTR + I + G model was used for subsequent phylogenetic analyses. BI analysis was performed in the program MrBayes v.3.2 (Ronquist F., Stockholm, Sweden) with a run of 10 million generations, and the average standard deviation of Bayesian split frequencies below 0.01 was considered to reach convergence [70]. The ML analysis was implemented in RaxML v.8.2 (Stamatakis A., Heidelberg, Germany) software with an evaluation of rapid inference for each node under 1000 ultrafast replications [71]. Data from the first 25% of the generations were removed as burn-in to improve the accuracy of the phylogenetic analysis results. Tracer v.1.7.1 (Drummond A.J., Edinburgh, UK) and FigTree v.1.4.0 (Rambaut A., Edinburgh, UK) were used to detect convergence to the stationary distribution of the chains and visualize the resulting trees, respectively [72,73].

### 2.5. Divergence Time Estimation

Fossil evidences of an evolutionary lineage might be able to calibrate the rate of evolution [74]. Fossil information can be used for phylogenetic analysis to find the minimum or maximum ages for the divergence time of internal nodes [75,76,77,78]. As such, four fossils were selected as the time-calibration points in this study. The first calibration point that we used belonged to Atalophlebiinae of Leptophlebiidae, with a divergence time of 15.00~20.00 Mya (the average is 17.50 Mya) [79,80]. The second calibration point was the divergence time in the genus *Ephemerella* of Ephemerellidae (41.30~47.80 Mya, 44.55 Mya average) [81], and the third time-calibration point was the first fossil record within Vietnamellidae (98.17~99.41 Mya, 98.79 Mya average) [82]. The fourth calibration point and the root time were set to the oldest ages of Ephemeroptera (168.82~239.51 Mya, 204.17 Mya average) and Odonata (221.00~450.00 Mya, 335.50 Mya), respectively [83]. Estimation of divergence time was mainly performed by the program MCMCTree in the PAML v.4.8 package and using the topology of the ML phylogenetic tree as the base tree [84]. The first step was to calculate the substitution rate, and then we calculated the gradient and Hessian of the branch lengths. Finally, MCMC was run to estimate the divergence time, and the parameters of the algorithm were set as burn-in period = 1,000,000, sample frequency = 1000 and the number of samples = 10,000. MCMC chains should be run at least twice from different starting points to check for convergence. The divergence time of the resulting tree was visualized in the FigTree v.1.4 program (Rambaut A., Edinburgh, UK) [73].

## 3. Results

### 3.1. Mitochondrial Genome Organization

**Figure 1:**
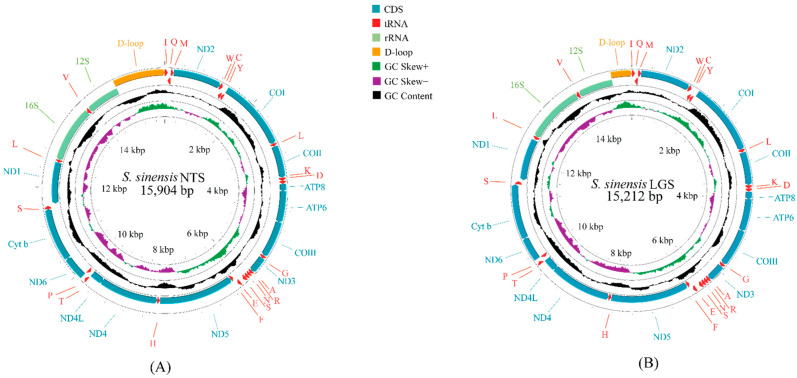
The mt genome maps of *S. chinensis* NTS (**A**) and *S. chinensis* LGS (**B**). The first circle represents the gene order. External genes are encoded by the positive strand, in contrast to the internal genes that are encoded by the negative strand. The second circle indicates the GC skew, and the third circle shows the GC content.

The complete mt genome of *S. chinensis* NTS was 15,904 bp in length, and the partial mt genome of *S. chinensis* LGS was 15,212 bp (Figure 1). Both mt genomes of *S. chinensis* species were circular double-stranded structures and contained the complete set of thirty-seven genes comprised thirteen PCGs, two rRNAs and twenty-two tRNAs, which were the same as the ancestral mt genome of Insecta (Tables S3 and S4). Among these 37 genes, twenty-three genes (fourteen tRNAs and nine PCGs) were located on the majority strand (H-strand), and the remaining fourteen genes (eight tRNAs, four PCGs and two rRNAs) were coded on the minority strand (L-strand) (Table S5). The total lengths of the 13 PCGs in *S. chinensis* NTS and *S. chinensis* LGS were 11,205 bp and 11,208 bp, respectively (Table 1). In these two newly sequenced mt genomes, all 13 PCGs used the typical start codon ATN (ATA/ATG/ATC/ATT). In the use of stop codons, ten PCGs stop codons in *S. chinensis* NTS were complete TAR (TAG/TAA), and the other three PCGs (COI, COII and ND5) used the incomplete stop codon T. By contrast, there were four PCGs (COI, COII, COIII and ND5) that used T as the stop codon in *S. chinensis* LGS (Table S5).

The AT skew, GC skew and A + T content of corresponding regions (whole genome, PCGs, rRNAs and tRNAs) of *S. chinensis* NTS and *S. chinensis* LGS were calculated and are shown in Table 1. The nucleotide composition of the *S. chinensis* NTS mt genome was A = 33.7%, T = 32.1%, C = 19.9% and G = 14.3%, which was very similar to that of *S. chinensis* LGS (A = 34.0%, T = 32.6%, C = 19.4% and G = 14.1%). Both the whole genome of *S. chinensis* NTS and *S. chinensis* LGS exhibited high A + T contents of 65.8% and 66.6%, and the GC skew was negative, whereas the AT skew was positive. We also observed that the A + T value of PCGs (−) was higher than PCGs (+) in both *S. chinensis* NTS and *S. chinensis* LGS.

**Figure 2:**
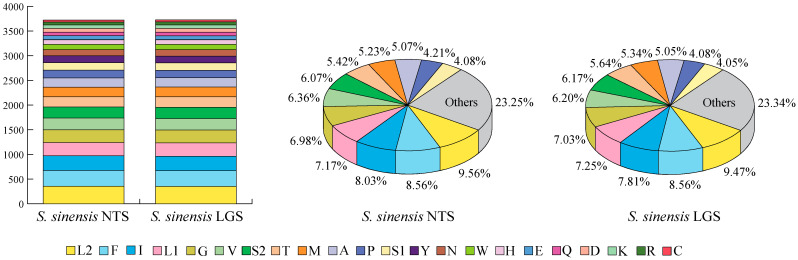
The number of different amino acids in the mt genomes of *S. chinensis* NTS and *S. chinensis* LGS. Different colors represent different amino acids. The left panel is a stacked histogram of the number of amino acid usages with decreasing frequency from bottom to top, and the right panel is a pie chart of the percentage of amino acid usages.

**Figure 3:**
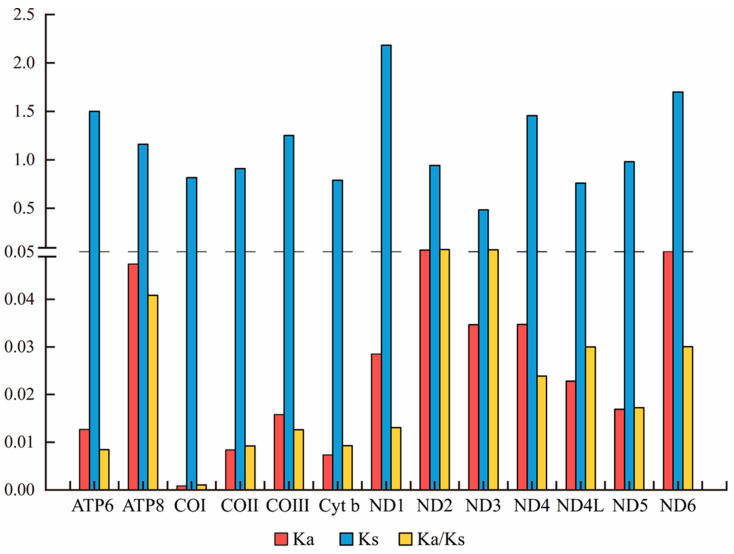
The values for Ka, Ks and Ka/Ks for *S. chinensis* NTS and *S. chinensis* LGS. The *y*-axis is broken at 0.05, and a change in *y*-axis numbering.

The amino acid numbers in the 13 PCGs of *S. chinensis* NTS and *S. chinensis* LGS are summarized in Figure 2. The overall codon usages were similar within the two newly sequenced mt genomes, with Leu1, Phe, Ile, Leu2 and Gly being the five most frequently coded amino acids (Figure 2). The relative synonymous codon usage (RSCU) in the PCGs of *S. chinensis* NTS, *S. chinensis* LGS and the two published mt genomes (*S. chinensis* HQ875717 and *S. chinensis* MF352165) was calculated, and the results are shown in Figure S1 and Table S6. In *S. chinensis* NTS and *S. chinensis* LGS, the total number of codons excluding stop codons were 3725 and 3726, respectively. Among the 62 codons of *S. chinensis* NTS, 27 codons were used more frequently (RSCU > 1), whereas 35 were less preferred codons (RSCU < 1) (Table S6). However, *S. chinensis* LGS had 35 codons with high frequency and 32 codons with low frequency. The most utilized codons in the 13 PCGs of the two mt genomes were UUA (Leu), AUU (Ile) and UUU (Phe) and were used ≥220 times. Calculated RSCU values showed that UUA (Leu) was the most frequently used among all codons, with an RSCU of 2.61 and 2.64 within *S. chinensis* NTS and *S. chinensis* LGS, respectively (Table S6). By contrast, codons with a third codon G or C were used very rarely; for instance, UCG (Ser), CCG (Pro), ACG (Thr), CGC (Arg) and AGG (Ser) had minimal usage (≤10 times) and AGG (Ser) was not used anytime in *S. chinensis* LGS. The ratio of Ka/Ks of each PCGs within *S. chinensis* NTS and *S. chinensis* LGS was calculated (Figure 3 and Table S7). The results showed that all PCGs had low Ka/Ks values (ω < 0.3), implying that all of them were under strong purifying selection. Among the 13 PCGs, the COI gene had the lowest ω value (0.001). Whereas, the ND2 gene had the highest ω value (0.074).

The length of 12S rRNA was 788 bp (*S. chinensis* NTS) and 778 bp (*S. chinensis* LGS), whereas 16S rRNA values were both 1286 bp in length. The A + T content of the rRNA genes of *S. chinensis* NTS and *S. chinensis* LGS was 68.2% and 69.4%, respectively (Table S5).

**Figure 4:**
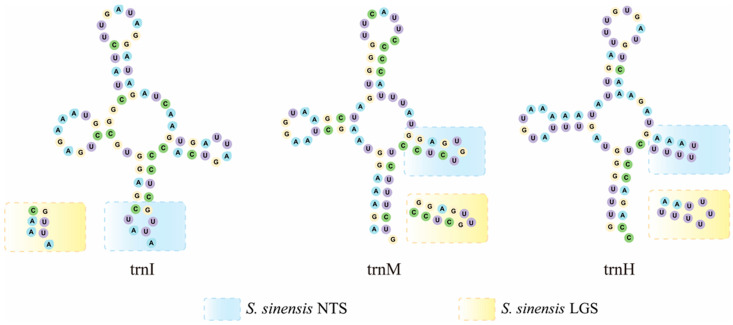
The secondary structures of the tRNA genes in *S. chinensis* NTS and *S. chinensis* LGS are shown with differences between the two species highlighted with boxes of different colors: *S. chinensis* NTS in blue and *S. chinensis* NTS in yellow.

The total length of the 22 tRNAs was 1431 bp (*S. chinensis* NTS) and 1430 bp (*S. chinensis* LGS). When comparing the secondary structures of the twenty-two tRNAs within the two species, three of them differed in their tRNA secondary structure (Figure 4). Of the 22 tRNA genes in the mt genomes of *S. chinensis* NTS and *S. chinensis* LGS, the secondary structure of most tRNA genes was identical and presented the normal cloverleaf model, except for trnI, trnM and trnH. Mismatches occurred in the acceptor stem of trnI in *S. chinensis* NTS, which was not present in *S. chinensis* LGS. Furthermore, a lack of the TΨC loop was observed in trnH and trnM among *S. chinensis* NTS and *S. chinensis* LGS, respectively. 

### 3.2. Calculation of Genetic Distance

The mt genomes of *S. chinensis* currently available in NCBI (HQ875717, MF352165) and *S. chinensis* NTS (ON729391) and *S. chinensis* LGS (ON729390) were used to explore the genetic distance between the four samples of the genus *Siphluriscus*. The results of comparing every genetic distance for each sample are presented in Table 2. The genetic distance of the four mt genomes of *S. chinensis* ranged from 0.2% to 12.3%, with an average of 6.27%. As presented in Table 2, a genetic distance of 0.2% occurred between *S. chinensis* LGS and *S.* *chinensis* MF352165, whereas a genetic distance of 0.3% occurred between *S. chinensis* LGS and *S.* *chinensis* HQ875717, suggesting that these three mt genomes belonged to the same species. By contrast, the *K2P* distance of *S. chinensis* NTS exhibited a high degree of diversity within Siphlonuridae. The genetic distances between *S. chinensis* NTS and *S. chinensis* HQ875717, and *S. chinensis* NTS and *S. chinensis* MF352165 were both 12.3%, reaching the species level. As discussed for species delimitation within *Siphluriscus*, the higher values observed in pairwise proportion of *S. chinensis* NTS and the other species supported the conclusion that *S. chinensis* NTS and *S. chinensis* LGS were not the same species.

### 3.3. Phylogenetic Analyses

Sequence heterogeneity analyses were performed first before proceeding with phylogeny, and the resulting AliGROOVE matrixes indicate the pairwise sequence comparisons of nucleotide datasets in all taxa with positive similarity scores (Figure S2). The AliGROOVE similarity scores shown in Figure S2 can detect whether there were sequences with high heterogeneity in the phylogenetic analysis, and the darker blue scores mean a non-randomized agreement within pairwise sequence comparison. The heterogeneity results from this study demonstrated that the pairwise sequence comparisons between the PCG12 and PCG123 datasets have a high degree of similarity (Figure S2). It can be seen from Figure S2 that the selected sequences have high similarity in both datasets and were suitable for further analysis. The heterogeneity of the PCG12 dataset was lower than that of the PCG123 dataset, so the PCG12 dataset was selected in the subsequent phylogenetic analyses.

**Figure 5:**
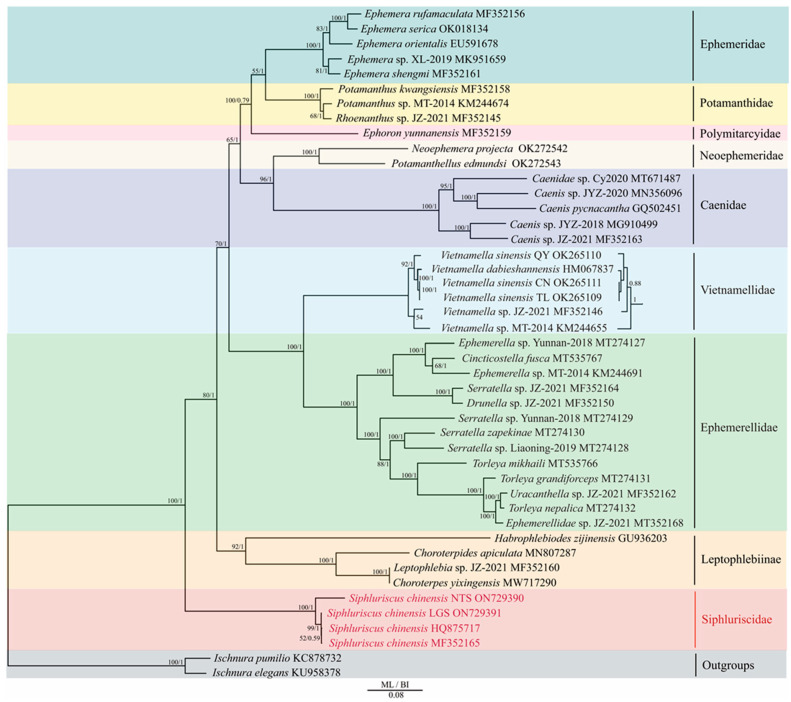
The ML (**left**) and BI (**right**) phylogenetic relationships among 45 species are based on the nucleotide dataset of the 13 mt PCGs. *Ischnura pumilio* (KC878732) and *Ischnura elegans* (KU958378) were used as the outgroups. The number on the left around each branch illustrates the bootstrap percentages of ML, whereas the number on the right indicates the posterior probability of BI. To the right of each species name is the GenBank accession number. The name of each Ephemeroptera family is listed on the right side of the figure and is distinguished by different colors. Species within Siphluriscidae are highlighted in red font.

Figure 5 shows the results for the analysis of the PCG12 datasets of 45 species. In addition, the ML and BI trees showed highly similar topological structures. The results of both ML and BI trees showed that Siphluriscidae was the oldest lineage within Ephemeroptera (Figure 5). Leptophlebiinae separated from the remaining families after Siphluriscidae. The clade of (((Ephemeridae + Potamanthidae) + Polymitarcyidae) + (Neophemeridae + Caenidae)) was a sister clade to the clade of (Ephemerellidae + Vietnamellidae). Focused on the phylogenetic relationship within Siphluriscidae, both the ML and BI trees showed a phylogenetic relationship of (((*S. chinensis* HQ875717 + *S. chinensis* MF352165) + *S. chinensis* LGS) + *S. chinensis* NTS).

### 3.4. Divergence Time Estimation

**Figure 6:**
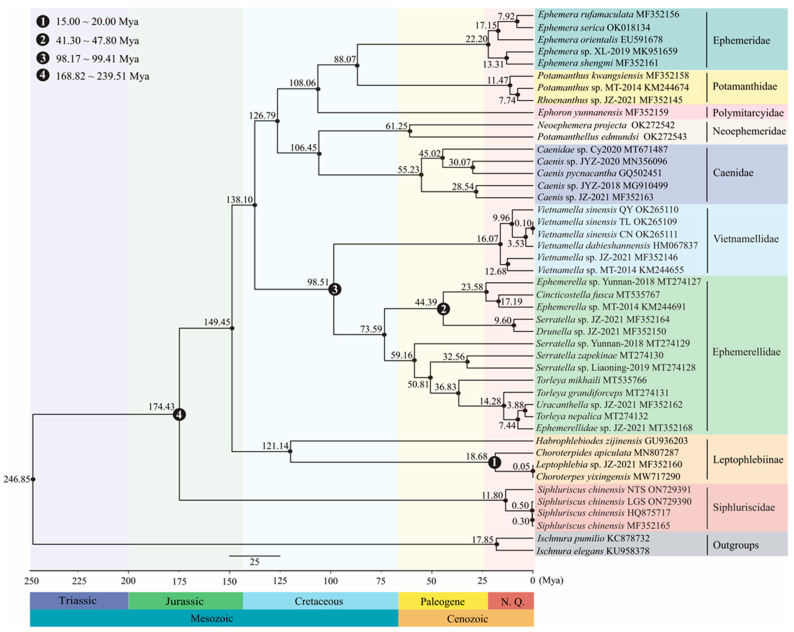
Evolutionary timescale within Ephemeroptera based on phylogenetic analyses. Median divergence times are provided above every node and the four fossil calibration points used are marked in the figure. The dates of each fossil calibration point are listed at the top left of the figure. The scale date is in million years ago (Mya) and the geological timescale is shown at the bottom of the chronogram.

This analysis estimated the divergence time among 43 Ephemeroptera species using four fossil calibration points based on the given tree topology in Figure 5. The mean divergence time and 95% highest posterior densities (HPD) range intervals of divergence times are shown in Figure 6 and Table 3. The root age of the tree dated to the Triassic, 246.85 million years ago (Mya) with 95% HPD = 177.44–364.11 Mya. The divergence times of this study suggested that most families within Ephemeroptera diversified during the Cretaceous era. Our estimated divergence dates using the calibrated substitution rate suggested that Siphluriscidae diverged from other families of Ephemeroptera in the Jurassic [174.43 Mya; 95% HPD = 163.38–197.39 Mya]. The results indicated that Leptophlebiinae occurred about 149.45 Mya [95% HPD = 129.50–171.22 Mya], and the most recent common ancestor (MRCA) of Vietnamellidae and Ephemerellidae diverged in the Cretaceous [98.51 Mya; 95% HPD = 98.00–99.00 Mya]. The MRCA of Neoephemeridae and Caenidae, 106.45 Mya [95% HPD = 83.84–129.98 Mya], is similar to the inferred MRCA of ((Ephemeridae + Potamanthidae) + Polymitarcyidae) [108.06 Mya; 95% HPD = 78.07–134.65 Mya]. Our results further indicated that the MRCA of ((Ephemeridae + Potamanthidae) + Polymitarcyidae) and (Vietnamellidae + Ephemerellidae) was estimated to be 126.79 Mya [95% HPD = 105.02–148.76 Mya]. The MRCA of four species within Siphluriscidae began to diversify during the Neogene [11.80 Mya; 95% HPD = 6.17–19.28 Mya], and *S. chinensis* NTS was first to diverge from the branches. After that, *S. chinensis* LGS was separated from *S. chinensis* (HQ875717) and *S. chinensis* (MF352165) at 0.50 Mya [95% HPD = 0.21–0.96 Mya]. Our divergence time estimation indicated that the MRCA of *S. chinensis* (HQ875717) and *S. chinensis* (MF352165) began to diversify at 0.30 Mya [95% HPD = 0.10–0.64 Mya].

## 4. Discussion

### 4.1. Composition Differences in Mitochondrial Genomes

Among the thirteen PCGs of *S. chinensis* HQ875717, *S. chinensis* MF352165 and *S. chinensis* LGS, nine PCGs used complete stop codons and four PCGs (COI, COII, COIII and ND5) used incomplete stop codons. However, only three PCGs (COI, COII and ND5) used incomplete stop codons in *S. chinensis* NTS. In both invertebrate and vertebrate mt genomes, the incomplete stop codons of PCGs are a common phenomenon [85,86,87,88]. Comparing the codon count and RSCU within four species showed a trend that the values between *S. chinensis* HQ875717, *S. chinensis* MF352165 and *S. chinensis* LGS differed slightly, whereas *S. chinensis* NTS showed a significant difference compared to the three other species (Table S6, Figure S1). Additionally, the average RSCU values of *S. chinensis* HQ875717, *S. chinensis* MF352165 and *S. chinensis* LGS were greater than or equal to one (RSCU ≥ 1), whereas *S. chinensis* NTS had average RSCU values of less than one (RSCU < 1). Among the 22 tRNA genes in the mt genomes of *S. chinensis* NTS and *S. chinensis* LGS, mismatches occurred in the acceptor stem of trnI in *S. chinensis* NTS, which was not present in *S. chinensis* LGS.

### 4.2. Phylogenetic Analyses and Identification of Cryptic Species

In order to assess the phylogenetic relationships within Ephemeroptera, we performed analyses using the 13 PCGs dataset (Figure 5). Based on different morphological characteristics and molecular data, some scholars regarded that Siphluriscidae as the most primitive of Ephemeroptera [1,5,6,7,8,23], whereas other scholars considered that Siphluriscidae is clustered into Ephemeroptera [9,10]. This divergence may be caused by the different selection of outgroups and families involved in the phylogenetic analysis.

In this study, ML and BI analyses produced well-supported phylogenetic trees where ((*S. chinensis* HQ875717 + *S. chinensis* MF352165) + *S. chinensis* LGS) was sister clade to *S. chinensis* NTS. We realized that *S. chinensis* NTS was distantly related to the above three species and had a distant phylogenetic placement within Siphluriscidae. In this study, the divergence time of Siphluriscidae was suggested to occur during the Jurassic period based on fossil and mt genome sequence data (Figure 6), which is consistent with previous findings [16]. After *S. chinensis* NTS diverged from the central nodes at around 11.80 Mya, *S. chinensis* LGS separated again from *S. chinensis* HQ875717 and *S. chinensis* MF352165 at around 0.50 Mya. The results similarly yielded a significant difference in divergence time between the three samples (*S. chinensis* HQ875717, *S. chinensis* MF352165 and *S. chinensis* LGS) and *S. chinensis* NTS, thus supporting the conclusion that *S. chinensis* HQ875717, *S. chinensis* MF352165 and *S. chinensis* LGS were the same species.

The pairwise genetic distance within *S. chinensis* HQ875717, *S. chinensis* MF352165 and *S. chinensis* LGS was relatively small, ranging from 0.2% (*S. chinensis* LGS–*S. chinensis* MF352165) to 0.3% (*S. chinensis* LGS–*S. chinensis* HQ875717) and (*S. chinensis* MF352165–*S. chinensis* HQ875717) (Table 2). By contrast, the genetic distances between *S. chinensis* NTS and the other three species was a high value, above 12.2% (*S. chinensis* NTS–*S. chinensis* MF352165). Except for the pairwise genetic distance within groups *S. chinensis* HQ875717, *S. chinensis* MF352165 and *S. chinensis* LGS, the other groups related to *S. chinensis* NTS were above 7% of regular insect reports [89]. Williams et al. found that the genetic distances of *Baetis rhodani* in different geographic locations was 8–19%, and then judged that some populations were cryptic species [90]. Based on these molecular data, the results indicate the existence of a cryptic species in *S. chinensis*. All three samples from Leigong Mountain (HQ875717, MF352165 and ON729390) belong to the same species, whereas the samples from Niutou Mountain (ON729391) belong to another species. Therefore, our study suggested that *S. chinensis* NTS was a cryptic species of *S. chinensis*.

## 5. Conclusions

Based on molecular analyses, a cryptic species belonging to Siphluriscidae was recognized. In this study, we successfully determined two newly sequenced mt genomes of *S. chinensis* NTS and *S. chinensis* LGS within Siphluriscidae, and we provided species delimitation of the *S. chinensis* complex based on a combination of genetic characteristics and genetic distance in the mt genome, phylogenetic relationship and divergence time. In combination with the collection sites, *S. chinensis* HQ875717, *S. chinensis* MF352165 and *S. chinensis* LGS were all collected from Guizhou Province, China, while *S. chinensis* NTS was collected from Zhejiang Province, China. The genetic distance between *S. chinensis* NTS and the other three species reached over 12.2%, which was higher than that of *S. chinensis* HQ875717, *S. chinensis* MF352165 and *S. chinensis* LGS, of 0.3%. BI and ML analyses indicated that *S. chinensis* NTS first separated from *S. chinensis* HQ875717, *S. chinensis* MF352165 and *S. chinensis* LGS at 11.80 Mya. Accordingly, it is highly probable that *S. chinensis* NTS was a cryptic species of *S. chinensis*, and the mt genome can be used as one of the effective molecular markers in the identification of cryptic species.

## Figures and Tables

**Table 1:** Composition of the mt genomes of *S. chinensis* NTS and *S. chinensis* LGS.

Region	Strand	*S. chinensis* NTS				*S. chinensis* LGS			
		Length (bp)	AT%	AT Skew	GC Skew	Length (bp)	AT%	AT Skew	GC Skew
Whole genome		15,904	65.8	0.025	−0.166	15,212	66.6	0.021	−0.159
PCGs	+	6876	63.6	−0.136	−0.178	6879	64.7	−0.145	−0.159
	-	4329	67.7	−0.256	0.197	4329	68.2	−0.244	0.190
tRNAs	+	908	66.2	−0.028	0.121	907	66.1	−0.010	0.111
	-	523	70.0	−0.055	0.312	523	70.4	−0.049	0.290
rRNAs	-	2074	68.2	−0.074	0.236	2064	69.4	−0.086	0.281

**Table 2:** The genetic distance of four mt genomes within Siphlonuridae.

	Species	GenBank No.	1	2	3
1	*S. chinensis*	HQ875717			
2	*S. chinensis*	MF352165	0.003		
3	*S. chinensis* LGS	ON729390	0.003	0.002	
4	*S. chinensis* NTS	ON729391	0.123	0.122	0.123

**Table 3:** Divergence times of nodes and clades within Ephemeroptera based on the mt genomes. All estimates are expressed as million years ago (Mya). The 95% highest posterior densities (HPD) are shown in the right column of the table. “&” represents the relationship between two branches.

Nodes/Clades	Mean Divergence Time (Mya)	95% HPD Range (Mya)
Ephemeridae & Potamanthidae	86.07	56.35~117.99
(Ephemeridae + Potamanthidae) & Polymitarcyidae	108.06	78.07~134.65
Neoephemeridae & Caenidae	106.45	83.84~129.98
(Neoephemeridae + Caenida) & (Ephemeridae + Potamanthidae+ Polymitarcyidae)	126.79	105.02~148.76
Vietnamellidae & Ephemerellidae	98.51	98.00~99.00
(Vietnamellidae + Ephemerellidae) & ((Neoephemeridae + Caenida) + ((Ephemeridae + Potamanthidae) + Polymitarcyidae))	138.10	119.64~159.06
Leptophlebiinae & ((Vietnamellidae + Ephemerellidae) + ((Neoephemeridae + Caenida) + ((Ephemeridae + Potamanthidae) + Polymitarcyidae)))	149.45	129.50~171.22
(Leptophlebiinae + ((Vietnamellidae + Ephemerellidae) + ((Neoephemeridae + Caenida) + ((Ephemeridae + Potamanthidae) + Polymitarcyidae)))) & Siphluriscidae	174.43	163.38~197.39

## Data Availability

The data supporting the findings of this study are openly available from the National Center for Biotechnology Information at https://www.ncbi.nlm.nih.gov (accessed on 10 June 2022). Accession numbers are: ON729390 and ON729391.

## Supplementary Materials

The following supporting information can be downloaded at: https://www.mdpi.com/article/10.3390/genes13101780/s1, Figure S1: The relative synonymous codon usage (RSCU) of the mt genome in *S. chinensis* NTS (A), *S. chinensis* LGS (B), *S. chinensis* HQ875717 (C) and *S. chinensis* MF352165 (D). The stop codons are not included; Figure S2: Heterogeneous sequence divergence within the 13 mt PCGs of 45 mt genomes for two datasets. (A) The PCG12 matrix dataset includes the first and second codon positions of the 13 PCGs; (B) the PCG123 matrix dataset includes the first, second and third codon positions of 13 the PCGs; Table S1: Sequences information used to reconstruct phylogenetic relationships; Table S2: The partition schemes and best-fitting models selected; Table S3: Location of features in the mt genome of *S. chinensis* NTS; Table S4: Location of features in the mt genome of *S. chinensis* LGS; Table S5: Features of the mt genomes of *S. chinensis* NTS (NTS) and *S. chinensis* LGS (LGS); Table S6: Codon counts and relative synonymous codon usage in the protein-coding genes of the mt genomes of *S. chinensis* NTS (SCN), *S. chinensis* LGS (SCL), *S. chinensis* HQ875717 (SCHQ) and *S. chinensis* MF352165 (SCMF); Table S7: The ratio of Ka/Ks for each gene of the 13 PCGs within *S. chinensis* NTS and *S. chinensis* LGS.

## Abbreviations

mt: mitochondrial; *S. chinensis* NTS: *Siphluriscus chinensis* collected from Niutou Mountain; *S. chinensis* LGS: *Siphluriscus chinensis* collected from Leigong Mountain; ML: maximum likelihood; BI: Bayesian inference; PCGs: protein-coding genes; tRNAs: transfer RNAs; rRNAs: ribosomal RNAs; NCBI: National Center for Biotechnology Information; K2P: Kimura-2 parameter; RSCU: relative synonymous codon usage; the PCG123 dataset: the first, second and third codons positions of the 13 PCGs; the PCG12 dataset: the first and second codons positions of the 13 PCGs; Mya: million years ago; HPD: highest posterior densities; MRCA: the most recent common ancestor.
