# Supplementary material for: The Genetic Diversity and the Divergence Time in Extant Primitive Mayfly, Siphluriscus chinensis Ulmer, 1920 Using the Mitochondrial Genome
Source: Genes (Basel). 2022 Oct 2;13(10):1780. doi: 10.3390/genes13101780 (PMC9601863; doi:10.3390/genes13101780)
Supplement: Supplementary file 1 [file genes-13-01780-s001.zip › TableS1. tree.pdf]

Table S1. Sequences information used to reconstruct phylogenetic relationships.

Family	Genus	Species	Length (bp)	GenBank No.	References
Caenidae	*Caenis*	*Caenis pycnacantha*	15,351	GQ502451	unpublished
Caenidae	*Caenis*	*Caenis* sp. JYZ-2018	15,254	MG910499	[8]
Caenidae	*Caenis*	*Caenis* sp. JYZ-2020	15,392	MN356096	[51]
Caenidae	*Caenis*	*Caenis* sp. JZ-2021	13,781	MF352163	[7]
Caenidae	unclassified	*Caenidae* sp. Cy2020	15,658	MT671487	[52]
Ephemerellidae	*Ephemerella*	*Ephemerella* sp. MT-2014	14,896	KM244691	[55]
Ephemerellidae	*Ephemerella*	*Ephemerella* sp. Yunnan-2018	15,256	MT274127	[23]
Ephemerellidae	*Serratella*	*Serratella* sp. Liaoning-2019	15,523	MT274128	[23]
Ephemerellidae	*Serratella*	*Serratella* sp. Yunnan-2018	15,134	MT274129	[23]
Ephemerellidae	*Serratella*	*Serratella zapekinae*	15,703	MT274130	[23]
Ephemerellidae	*Serratella*	*Serratella* sp. JZ-2021	16,751	MF352164	[7]
Ephemerellidae	*Torleya*	*Torleya mikhaili*	15,042	MT535766	[53]
Ephemerellidae	*Torleya*	*Torleya nepalica*	15,599	MT274132	[23]
Ephemerellidae	*Torleya*	*Torleya grandiforceps*	15,330	MT274131	[23]
Ephemerellidae	*Drunella*	*Drunella* sp. JZ-2021	15,128	MF352150	[7]
Ephemerellidae	*Uracanthella*	*Uracanthella* sp. JZ-2021	15,160	MF352162	[7]
Ephemerellidae	*Cincticostella*	*Cincticostella fusca*	15,135	MT535767	[53]
Ephemerellidae	unclassified	*Ephemerellidae* sp. JZ-2021	15,154	MF352168	[7]
Ephemeridae	*Ephemera*	*Ephemera rufomaculata*	14,451	MF352156	[7]
Ephemeridae	*Ephemera*	*Ephemera shengmi*	15,149	MF352161	[7]
Ephemeridae	*Ephemera*	*Ephemera. orientalis*	16,463	EU591678	[54]
Ephemeridae	*Ephemera*	*Ephemera* sp. XL-2019	15,314	MK951659	[6]
Ephemeridae	*Ephemera*	*Ephemera serica*	15,004	OK018134	[60]
Leptophlebiidae	*Choroterpides*	*Choroterpides apiculata*	15,199	MN807287	[58]
Leptophlebiidae	*Habrophlebiodes*	*Habrophlebiodes zijinensis*	14,355	GU936203	unpublished
Leptophlebiidae	*Leptophlebia*	*Leptophlebia* sp. JZ-2021	15,534	MF352160	[7]
Leptophlebiidae	*Atalophlebiinae*	*Choroterpes yixingensis*	15,534	MW717290	[59]
Neoephemeridae	*Neoephemera*	*Neoephemera projecta*	16,031	OK272542	[53]
Neoephemeridae	*Neoephemera*	*Potamanthellus edmundsi*	15,274	OK272543	[53]
Polymitarcyidae	*Ephoron*	*Ephoron yunnanensis*	13,949	MF352159	[7]
Potamanthidae	*Potamanthus*	*Potamanthus* sp. MT-2014	14,937	KM244674	[55]
Potamanthidae	*Potamanthus*	*Rhoenanthus* sp. JZ-2021	14,118	MF352145	[7]
Potamanthidae	*Potamanthus*	*Potamanthus kwangsiensis*	13,988	MF352158	[7]
Siphluriscidae	*Siphluriscus*	*Siphluriscus chinensis*	16,616	HQ875717	[5]
Siphluriscidae	*Siphluriscus*	*Siphluriscus chinensis*	14,424	MF352165	[7]
Siphluriscidae	*Siphluriscus*	*Siphluriscus chinensis* NTS	15,999	ON729390	this study
Siphluriscidae	*Siphluriscus*	*Siphluriscus chinensis* LGS	15,212	ON729391	this study
Vietnamellidae	*Vietnamella*	*Vietnamella* sp. MT-2014	15,043	KM244655	[55]
Vietnamellidae	*Vietnamella*	*Vietnamella dabieshanensis*	15,761	HM067837	unpublished
Vietnamellidae	*Vietnamella*	*Vietnamella* sp. JZ-2021	16,006	MF352146	[7]
Vietnamellidae	*Vietnamella*	*Vietnamella sinensis* TL	15,674	OK265109	[57]
Vietnamellidae	*Vietnamella*	*Vietnamella sinensis* QY	15,610	OK265110	[57]
Vietnamellidae	*Vietnamella*	*Vietnamella sinensis* CN	15,674	OK265111	[57]
Coenagrionidae	*Ischnura*	*Ischnura pumilio*	15,250	KC878732	[61]
Coenagrionidae	*Ischnura*	*Ischnura elegans*	15,962	KU958378	[62]
