# Supplementary material for: The Genetic Diversity and the Divergence Time in Extant Primitive Mayfly, Siphluriscus chinensis Ulmer, 1920 Using the Mitochondrial Genome
Source: Genes (Basel). 2022 Oct 2;13(10):1780. doi: 10.3390/genes13101780 (PMC9601863; doi:10.3390/genes13101780)
Supplement: Supplementary file 1 [file genes-13-01780-s001.zip › TableS2. partition.pdf]

Table S2. The partition schemes and best-fitting models selected.

Nucleotide Sequence Alignments		
Subset	Subset Partitions	Best Model
Partition 1	Cyt b_pos1, ATP6_pos1	GTR+I+G
Partition 2	COI_pos2, COII_pos2, COIII_pos2, Cyt b_pos2, ATP6_pos2	TVM+I+G
Partition 3	ND2_pos1, ATP8_pos1, ND6_pos1, ND3_pos1	GTR+I+G
Partition 4	ATP8_pos2, ND3_pos2, ND2_pos2, ND6_pos2	GTR+I +G
Partition 5	COI_pos1, COII_pos1, COIII_pos1	GTR+I+G
Partition 6	ND4L_pos1, ND4_pos1, ND5_pos1, ND1_pos1	GTR+I+G
Partition 7	ND1_pos2, ND5_pos2, ND4_pos2, ND4L_pos2	GTR+I+G
