# Supplementary material for: The Genetic Diversity and the Divergence Time in Extant Primitive Mayfly, Siphluriscus chinensis Ulmer, 1920 Using the Mitochondrial Genome
Source: Genes (Basel). 2022 Oct 2;13(10):1780. doi: 10.3390/genes13101780 (PMC9601863; doi:10.3390/genes13101780)
Supplement: Supplementary file 1 [file genes-13-01780-s001.zip › TableS3. NTS location.pdf]

Table S3. Location of features in the mt genome of *Siphluriscus chinensis* NTS.

Gene	Strand	Position	Length (nuc.)	Anti Codon	Start Codon	Stop Codon	Intergenic nucleotides
$tRNA^{Ile}$	+	1-64	64	ATC			-3
$tRNA^{Gln}$	-	62-130	69	CAA			-1
$tRNA^{Met}$	+	130-193	64	ATG			0
ND2	+	194-1228	1035		ATT	TAA	-2
$tRNA^{Trp}$	+	1227-1292	66	TGA			-8
$tRNA^{Cys}$	-	1285-1346	62	TGC			0
$tRNA^{Tyr}$	-	1347-1411	65	TAC			-8
COI	+	1404-2943	1540		ATT	T	0
$tRNA^{Leu2}$	+	2944-3008	65	TTA			+4
COII	+	3013-3700	688		ATG	T	0
$tRNA^{Lys}$	+	3701-3769	69	AAG			0
$tRNA^{Asp}$	+	3770-3835	66	GAC			0
ATP8	+	3836-3994	159		ATT	TAA	-4
ATP6	+	3991-4662	672		ATA	TAA	-1
COIII	+	4662-5447	786		ATG	TAG	+2
$tRNA^{Gly}$	+	5450-5513	64	GGA			0
ND3	+	5514-5867	354		ATT	TAG	-2
$tRNA^{Ala}$	+	5866-5928	63	GCA			-1
$tRNA^{Arg}$	+	5928-5988	61	CGA			-1
$tRNA^{Asn}$	+	5988-6051	64	AAC			0
$tRNA^{Ser1}$	+	6052-6118	67	AGC			+85
$tRNA^{Glu}$	+	6204-6267	64	GAA			0
$tRNA^{Phe}$	-	6268-6331	64	TTC			-3
ND5	-	6329-8063	1735		ATT	T	0
$tRNA^{His}$	-	8064-8126	63	CAC			-1
ND4	-	8126-9478	1353		ATT	TAG	-13
ND4L	-	9466-9762	297		ATG	TAA	+2
$tRNA^{Thr}$	+	9765-9826	62	ACA			0
$tRNA^{Pro}$	-	9827-9890	64	CCA			+2
ND6	+	9893-10399	507		ATT	TAA	-1
Cyt *b*	+	10399-11535	1137		ATG	TAA	-2
$tRNA^{Ser2}$	+	11534-11602	69	TCA			+29
ND1	-	11632-12576	945		TTG	TAA	+1
$tRNA^{Leu1}$	-	12578-12642	65	CTA			0
16S rRNA	-	12643-13928	1286				0
$tRNA^{Val}$	-	13929-13999	71	GTA			0
12S rRNA	-	14000-14787	788				0
CR	+	14788-15904	1117				
