# Supplementary material for: The Genetic Diversity and the Divergence Time in Extant Primitive Mayfly, Siphluriscus chinensis Ulmer, 1920 Using the Mitochondrial Genome
Source: Genes (Basel). 2022 Oct 2;13(10):1780. doi: 10.3390/genes13101780 (PMC9601863; doi:10.3390/genes13101780)
Supplement: Supplementary file 1 [file genes-13-01780-s001.zip › TableS4. LGS location.pdf]

Table S4. Location of features in the mt genome of *Siphluriscus chinensis* LGS.

Gene	Strand	Position	Length (nuc.)	Anti Codon	Start Codon	Stop Codon	Intergenic nucleotides
$tRNA^{Ile}$	+	1-64	64	ATC			-3
$tRNA^{Gln}$	-	62-130	69	CAA			-1
$tRNA^{Met}$	+	130-193	64	ATG			0
ND2	+	194-1228	1035		ATT	TAA	-2
$tRNA^{Trp}$	+	1227-1292	66	TGA			-8
$tRNA^{Cys}$	-	1285-1346	62	TGC			0
$tRNA^{Tyr}$	-	1347-1411	65	TAC			-8
COI	+	1404-2943	1540		ATT	T	0
$tRNA^{Leu2}$	+	2944-3008	65	TTA			+4
COII	+	3013-3700	688		ATG	T	0
$tRNA^{Lys}$	+	3701-3769	69	AAG			0
$tRNA^{Asp}$	+	3770-3834	65	GAC			+1
ATP8	+	3836-3994	159		ATT	TAA	-7
ATP6	+	3988-4662	675		ATA	TAA	-1
COIII	+	4662-5448	787		ATG	T	0
$tRNA^{Gly}$	+	5449-5512	64	GGA			0
ND3	+	5513-5866	354		ATT	TAG	-2
$tRNA^{Ala}$	+	5865-5927	63	GCA			-1
$tRNA^{Arg}$	+	5927-5987	61	CGA			-1
$tRNA^{Asn}$	+	5987-6050	64	AAC			0
$tRNA^{Ser1}$	+	6051-6117	67	AGC			+85
$tRNA^{Glu}$	+	6203-6266	64	GAA			0
$tRNA^{Phe}$	-	6267-6330	64	TTC			-3
ND5	-	6328-8062	1735		ATT	T	0
$tRNA^{His}$	-	8063-8125	63	CAC			-1
ND4	-	8125-9477	1353		ATT	TAG	-13
ND4L	-	9465-9761	297		ATG	TAA	+2
$tRNA^{Thr}$	+	9764-9825	62	ACA			0
$tRNA^{Pro}$	-	9826-9889	64	CCA			+2
ND6	+	9892-10398	507		ATT	TAA	-1
Cyt *b*	+	10398-11534	1137		ATG	TAA	-2
$tRNA^{Ser2}$	+	11533-11601	69	TCA			+30
ND1	-	11632-12576	945		ATT	TAA	+1
$tRNA^{Leu1}$	-	12578-12642	65	CTA			0
16S rRNA	-	12643-13928	1286				0
$tRNA^{Val}$	-	13929-13999	71	GTA			0
12S rRNA	-	14000-14777	778				0
CR	+	14778-15212	435				
