# Supplementary material for: The Genetic Diversity and the Divergence Time in Extant Primitive Mayfly, Siphluriscus chinensis Ulmer, 1920 Using the Mitochondrial Genome
Source: Genes (Basel). 2022 Oct 2;13(10):1780. doi: 10.3390/genes13101780 (PMC9601863; doi:10.3390/genes13101780)
Supplement: Supplementary file 1 [file genes-13-01780-s001.zip › TableS5. features.pdf]

Table S5. Features of the mt genomes of *S. chinensis* NTS (NTS) and *S. chinensis* LGS (LGS).

Gene	Strand	Position		Intergenic Nucleotides		Codon		Anticodon
		NTS	LGS	NTS	LGS	NTS	LGS	
tRNA^{Ile}	+	1-64	1-64	0	0			ATC
tRNA^{Gln}	-	62-130	62-130	-3	-3			CAA
tRNA^{Met}	+	130-193	130-193	-1	-1			ATG
ND2	+	194-1228	194-1228	0	0	ATT/TAA	ATT/TAA	
tRNA^{Trp}	+	1227-1292	1227-1292	-2	-2			TGA
tRNA^{Cys}	-	1285-1346	1285-1346	-8	-8			TGC
tRNA^{Tyr}	-	1347-1411	1347-1411	0	0			TAC
COI	+	1404-2943	1404-2943	-8	-8	ATT/T	ATT/T	
tRNA^{Leu2}	+	2944-3008	2944-3008	0	0			TTA
COII	+	3013-3700	3013-3700	+4	+4	ATG/T	ATG/T	
tRNA^{Lys}	+	3701-3769	3701-3769	0	0			AAG
tRNA^{Asp}	+	3770-3835	3770-3834	0	0			GAC
ATP8	+	3836-3994	3836-3994	0	+1	ATT/TAA	ATT/TAA	
ATP6	+	3991-4662	3988-4662	-4	-7	ATA/TAA	ATA/TAA	
COIII	+	4662-5447	4662-5448	-1	-1	ATG/TAG	ATG/T	
tRNA^{Gly}	+	5450-5513	5449-5512	+2	0			GGA
ND3	+	5514-5867	5513-5866	0	0	ATT/TAG	ATT/TAG	
tRNA^{Ala}	+	5866-5928	5865-5927	-2	-2			GCA
tRNA^{Arg}	+	5928-5988	5927-5987	-1	-1			CGA
tRNA^{Asn}	+	5988-6051	5987-6050	-1	-1			AAC
tRNA^{Ser1}	+	6052-6118	6051-6117	0	0			AGC
tRNA^{Glu}	+	6204-6267	6203-6266	+85	+85			GAA
tRNA^{Phe}	-	6268-6331	6267-6330	0	0			TTC
ND5	-	6329-8063	6328-8062	-3	-3	ATT/T	ATT/T	
tRNA^{His}	-	8064-8126	8063-8125	0	0			CAC
ND4	-	8126-9478	8125-9477	-1	-1	ATT/TAG	ATT/TAG	
ND4L	-	9466-9762	9465-9761	-13	-13	ATG/TAA	ATG/TAA	
tRNA^{Thr}	+	9765-9826	9764-9825	+2	+2			ACA
tRNA^{Pro}	-	9827-9890	9826-9889	0	0			CCA
ND6	+	9893-10399	9892-10398	+2	+2	ATT/TAA	ATT/TAA	
Cyt *b*	+	10399-11535	10398-11534	-1	-1	ATG/TAA	ATG/TAA	
tRNA^{Ser2}	+	11534-11602	11533-11601	-2	-2			TCA
ND1	-	11632-12576	11632-12576	+29	+30	TTG/TAA	ATT/TAA	
tRNA^{Leu1}	-	12578-12642	12578-12642	+1	+1			CTA
16S rRNA	-	12643-13928	12643-13928	0	0			
tRNA^{Val}	-	13929-13999	13929-13999	0	0			GTA
12S rRNA	-	14000-14787	14000-14777	0	0			
CR	+	14788-15904	14778-15212	0	0			
