# Supplementary material for: The Genetic Diversity and the Divergence Time in Extant Primitive Mayfly, Siphluriscus chinensis Ulmer, 1920 Using the Mitochondrial Genome
Source: Genes (Basel). 2022 Oct 2;13(10):1780. doi: 10.3390/genes13101780 (PMC9601863; doi:10.3390/genes13101780)
Supplement: Supplementary file 1 [file genes-13-01780-s001.zip › TableS6. RSCU.pdf]

Table S6. Codon counts and relative synonymous codon usage in the protein coding genes of the mt genomes of *S. chinensis* NTS (SCN), *S. chinensis* LGS (SCL), *S. chinensis* HQ875717 (SCHQ) and *S. chinensis* MF352165 (SCMF).

Codon	Count				RSCU				Codon	Count				RSCU			
	SCN	SCL	SCHQ (HQ875717)	SCMF (MF352165)	SCN	SCL	SCHQ (HQ875717)	SCMF (MF352165)		SCN	SCL	SCHQ (HQ875717)	SCMF (MF352165)	SCN	SCL	SCHQ (HQ875717)	SCMF (MF352165)
UUU(F)	220	223	223	221	1.38	1.40	1.40	1.39	GCG(A)	10	12	13	13	0.21	0.26	0.28	0.28
UUC(F)	99	96	95	98	0.62	0.60	0.60	0.61	UAU(Y)	93	102	102	103	1.35	1.48	1.48	1.48
UUA(L)	271	274	274	271	2.61	2.64	2.64	2.63	UAC(Y)	45	36	36	36	0.65	0.52	0.52	0.52
UUG(L)	85	79	78	79	0.82	0.76	0.75	0.77	CAU(H)	54	42	44	42	1.14	0.89	0.94	0.89
CUU(L)	114	116	116	115	1.10	1.12	1.12	1.12	CAC(H)	41	52	50	52	0.86	1.11	1.06	1.11
CUC(L)	40	38	39	38	0.39	0.37	0.38	0.37	CAA(Q)	55	60	62	60	1.51	1.60	1.65	1.6
CUA(L)	91	92	91	92	0.88	0.89	0.88	0.89	CAG(Q)	18	15	13	15	0.49	0.40	0.35	0.4
CUG(L)	22	24	25	23	0.21	0.23	0.24	0.22	AAU(N)	92	96	97	95	1.44	1.48	1.49	1.48
AUU(I)	228	234	234	233	1.53	1.61	1.61	1.61	AAC(N)	36	34	33	33	0.56	0.52	0.51	0.52
AUC(I)	71	57	57	57	0.47	0.39	0.39	0.39	AAA(K)	34	36	36	35	0.97	1.00	1.00	0.99
AUA(M)	133	144	147	145	1.36	1.45	1.46	1.46	AAG(K)	36	36	36	36	1.03	1.00	1.00	1.01
AUG(M)	62	55	54	54	0.64	0.55	0.54	0.54	GAU(D)	51	53	54	52	1.40	1.45	1.48	1.44
GUU(V)	87	94	94	94	1.47	1.63	1.63	1.63	GAC(D)	22	20	19	20	0.60	0.55	0.52	0.56
GUC(V)	20	14	14	15	0.34	0.24	0.24	0.26	GAA(E)	67	66	66	65	1.61	1.63	1.63	1.6
GUA(V)	92	91	90	90	1.55	1.58	1.56	1.57	GAG(E)	16	15	15	16	0.39	0.37	0.37	0.4
GUG(V)	38	32	33	31	0.64	0.55	0.57	0.54	UGU(C)	33	35	35	35	1.53	1.71	1.71	1.71
UCU(S)	117	125	124	124	2.48	2.62	2.58	2.60	UGC(C)	10	6	6	6	0.47	0.29	0.29	0.29
UCC(S)	40	36	39	36	0.85	0.75	0.81	0.75	UGA(W)	85	89	88	88	1.65	1.70	1.68	1.69
UCA(S)	60	59	58	59	1.27	1.24	1.21	1.24	UGG(W)	18	16	17	16	0.35	0.30	0.32	0.31
UCG(S)	9	10	11	10	0.19	0.21	0.23	0.21	CGU(R)	21	24	24	24	1.40	1.57	1.57	1.57
CCU(P)	63	80	82	80	1.61	2.12	2.17	2.12	CGC(R)	3	6	6	6	0.20	0.39	0.39	0.39
CCC(P)	50	30	28	30	1.27	0.79	0.74	0.79	CGA(R)	26	27	28	28	1.73	1.77	1.84	1.84
CCA(P)	37	31	31	31	0.94	0.82	0.82	0.82	CGG(R)	10	4	3	3	0.67	0.26	0.20	0.2
CCG(P)	7	10	10	10	0.18	0.26	0.26	0.26	AGU(S)	41	57	57	55	0.87	1.19	1.19	1.15
ACU(T)	102	106	106	105	2.02	2.02	2.02	2.01	AGC(S)	19	12	12	14	0.40	0.25	0.25	0.29
ACC(T)	40	33	34	35	0.79	0.63	0.65	0.67	AGA(S)	90	83	83	84	1.90	1.74	1.73	1.76
ACA(T)	51	66	65	65	1.01	1.26	1.24	1.24	AGG(S)	2	0	0	0	0.04	0.00	0.00	0
ACG(T)	9	5	5	4	0.18	0.10	0.10	0.08	GGU(G)	49	52	53	56	0.75	0.79	0.81	0.86
GCU(A)	90	85	84	84	1.90	1.81	1.80	1.80	GGC(G)	30	20	20	18	0.46	0.31	0.31	0.28
GCC(A)	45	44	44	44	0.95	0.94	0.94	0.94	GGA(G)	119	122	124	118	1.83	1.86	1.89	1.82
GCA(A)	44	47	46	46	0.93	1.00	0.98	0.98	GGG(G)	62	68	65	68	0.95	1.04	0.99	1.05
