# Supplementary material for: The Genetic Diversity and the Divergence Time in Extant Primitive Mayfly, Siphluriscus chinensis Ulmer, 1920 Using the Mitochondrial Genome
Source: Genes (Basel). 2022 Oct 2;13(10):1780. doi: 10.3390/genes13101780 (PMC9601863; doi:10.3390/genes13101780)
Supplement: Supplementary file 1 [file genes-13-01780-s001.zip › TableS7. KaKs.pdf]

Table S7. The ratio of Ka/Ks for each gene of the 13 PCGs within *S. chinensis* NTS and *S. chinensis* LGS.

PCGs	Ka	Ks	Ka/Ks
ATP6	0.0126468	1.49913	0.0084361
ATP8	0.0474415	1.16035	0.0408854
COI	0.000849998	0.817454	0.00103981
COII	0.00837904	0.910273	0.00920497
COIII	0.0157606	1.25303	0.012578
Cyt b	0.00732095	0.789105	0.00927753
ND1	0.028493	2.18433	0.0130443
ND2	0.0699011	0.943615	0.074078
ND3	0.0346585	0.484431	0.0715448
ND4	0.0347507	1.45559	0.023874
ND4L	0.0228176	0.760729	0.0299943
ND5	0.0169	0.980598	0.0172344
ND6	0.051018	1.69937	0.0300218
