# Supplementary figures and images for: The Genetic Diversity and the Divergence Time in Extant Primitive Mayfly, Siphluriscus chinensis Ulmer, 1920 Using the Mitochondrial Genome
Source: Genes (Basel). 2022 Oct 2;13(10):1780. doi: 10.3390/genes13101780 (PMC9601863; doi:10.3390/genes13101780)

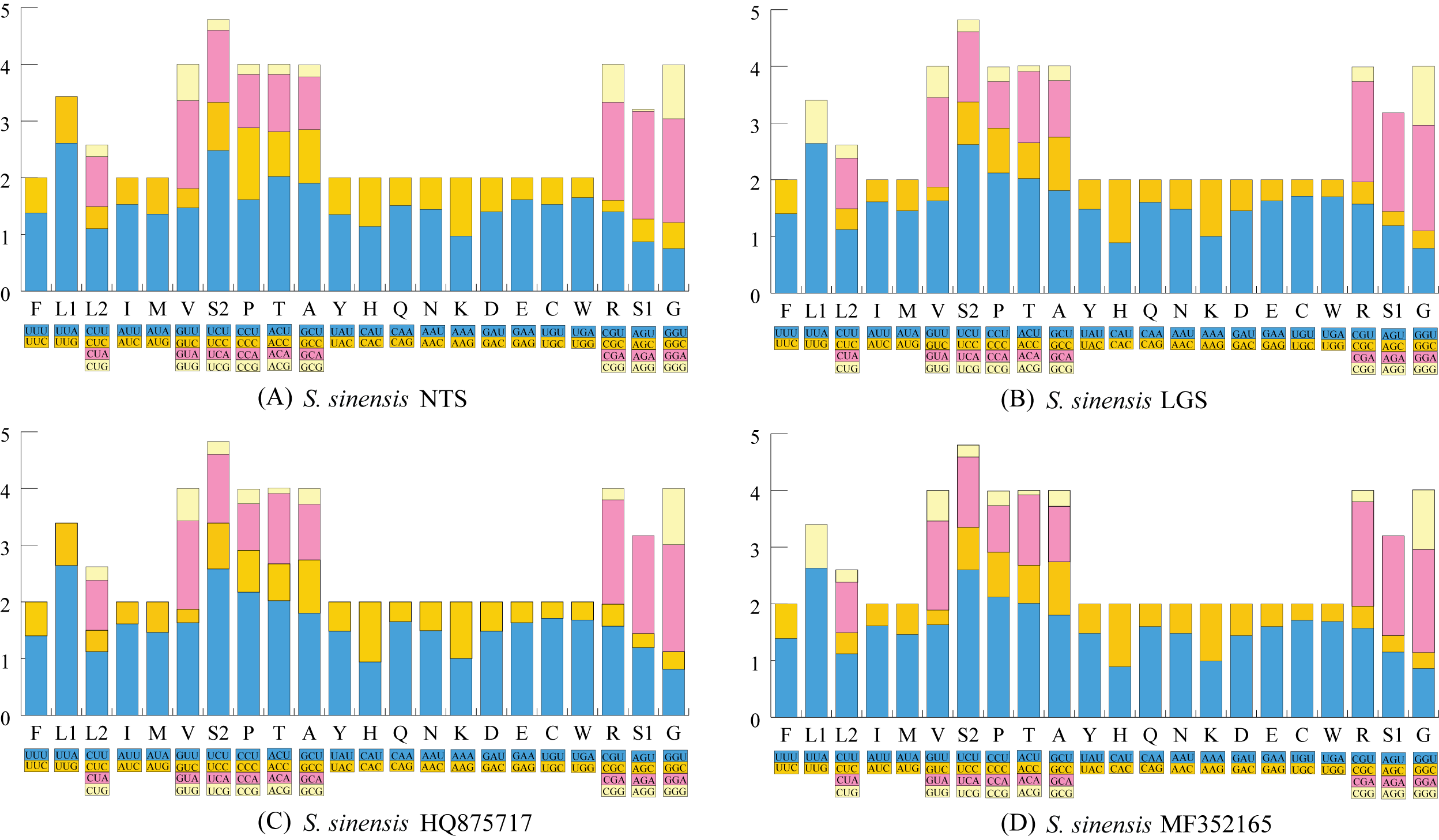

Supplement: Supplementary file 1 [file genes-13-01780-s001.zip › FigureS1. RSCU.png]

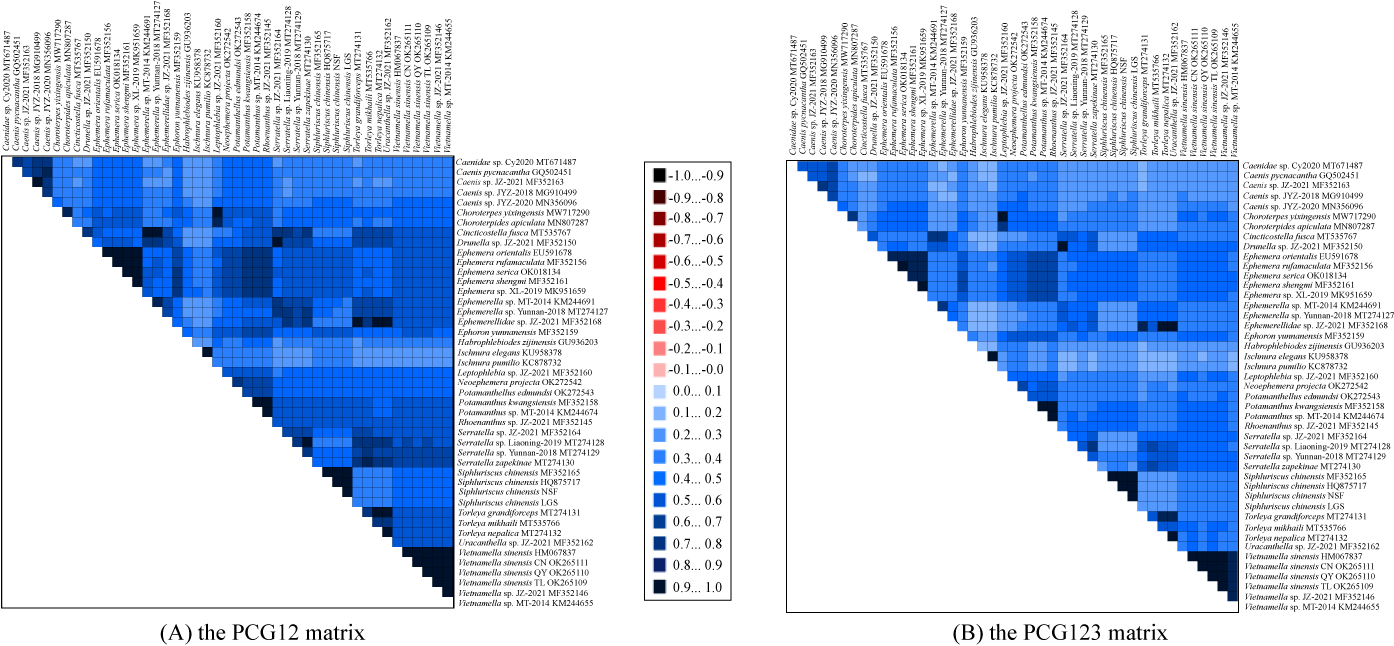

Supplement: Supplementary file 1 [file genes-13-01780-s001.zip › FigureS2. AliGROOVE.png]
